# Activity Fractionation Moderates the Relationship of Gait Speed With Alzheimer’s Disease Risk

**DOI:** 10.1093/geroni/igab046.614

**Published:** 2021-12-17

**Authors:** Yang An, Jennifer Schrack, Pei-Lun Kuo, Amal Wanigatunga, Eleanor Simonsick, Susan Resnick, Luigi Ferrucci, Qu Tian

**Affiliations:** 1 NIA, Baltimore, Maryland, United States; 2 Johns Hopkins Bloomberg School of Public Health, Baltimore, Maryland, United States; 3 National Institute on Aging, National Institute on Aging, Maryland, United States; 4 National Instute on Aging/NIH, Baltimore, Maryland, United States; 5 National Institute on Aging, Baltimore, Maryland, United States

## Abstract

Older adults with slow gait have a modestly elevated risk of Alzheimer’s disease (AD). Whether strategies to maintain function, such as interlacing periods of activity and rest, modify this relationship is unknown. We analyzed 577 initially cognitively normal participants aged 50+(53%women,26%Black) who had baseline data on gait speed and fractionation via ActiHeart. Diagnoses of mild cognitive impairment (MCI)/AD were adjudicated during an average 7.3years follow-up. We examined gait speed, fractionation, and their interaction with MCI/AD risk using Cox proportional-hazards models, adjusted for demographics and APOE-e4. Each 0.2m/sec faster gait speed was associated with 24% lower risk of MCI/AD(p=0.04). Fractionation was not associated with MCI/AD risk(p>0.05). There was a significant gait*fractionation interaction(p=0.013). At high fractionation, gait was not predictive of MCI/AD. Slow gait speed is less predictive of future MCI/AD in individuals who fractionate their activity to maintain function, possibly indicating brain function that drives such compensatory strategy is still conserved.

